# 2StrucCompare: a webserver for visualizing small but noteworthy differences between protein tertiary structures through interrogation of the secondary structure content

**DOI:** 10.1093/nar/gkz456

**Published:** 2019-05-22

**Authors:** Elliot D Drew, Robert W Janes

**Affiliations:** School of Biological and Chemical Sciences, Queen Mary University of London, Mile End Road, London, E1 4NS, UK

## Abstract

2StrucCompare is a webserver whose primary aim is to visualize subtle but functionally important differences between two related protein structures, either of the same protein or related homologues, with similar or functionally different tertiary structures. At the heart of the package is identifying and visualizing differences between conformations at the secondary structure and at the residue level, such as contact differences or side chain conformational differences found between two protein chains. The protein secondary structures are determined according to four established methods (DSSP, STRIDE, P-SEA and STICKS), and as each employs different assignment strategies, small conformational differences between the two structures can give rise to paired residues being denoted as having different secondary structure features with the different methods. 2StrucCompare captures both the large and more subtle differences found between structures, enabling visualization of these differences that could be key to an understanding of a proteins’ function.

2StrucCompare is freely accessible at http://2struccompare.cryst.bbk.ac.uk/index.php

## INTRODUCTION

When examining the structure of a protein, it is often a key step in this process to make some conformational comparisons to other protein structures. Many reasons exist for such comparisons to be made. Examples include examining the similarities between homologous proteins from different species; establishing the degree of similarity between a predicted and experimentally-determined structure; identifying the effects of mutations, either introduced or naturally-occurring, on a structure relative to the wild-type conformation; exploring the relationship between structures with and without ligands/effector molecules being bound; or even exploring the effects on a protein structure where one of a pair is the ‘dark structure’ and the other has been irradiated to induce a conformational change in a chromophore bound into it. The utilization of graphics packages substantially aids this structure comparison process.

A variety of computational/graphics methods exist online for the comparison of protein structures. These include the Research Collaboratory for Structural Bioinformatics Protein Data Bank (RCSB PDB) Protein Comparison Tool (1) that provides a range of sequence and structural alignment methods, pairwise structural alignment servers like DALI (2), TMalign (3) and SuperPose (4) and databases of pre-calculated aligned structures, included in VAST (5) and VAST+ (6) that allow for visualization of query proteins aligned with structurally similar molecules. In addition, some downloadable molecular visualization packages such as PyMol (7), VMD (8) and MolMol (9) also enable the superposition of proteins, usually calculating the alignment quality by the root mean square deviation (RMSD) between the two structures. These online services and downloadable packages are optimized for fold comparison, where it is primarily the reporting of the minimal differences between the polypeptide backbone atoms that is under consideration; how ‘similar’ are the two proteins? There is no package, however, that first optimizes the alignment between two protein chains, and then specifically examines the ‘differences’ between these chains at the more focussed secondary structure and intra-residue contact level. For this reason, we have created 2StrucCompare.

### THE 2StrucCompare SERVER

The aims of the 2StrucCompare server are to enable a user to identify conformationally different and/or significantly interesting features between a pair of highly similar protein chains, with the differences presented in both a visually informative and tabulated format. Here ‘similar’ could refer to the pair being identical in sequences, but with differing overall conformations, or could refer to two structurally similar proteins with different sequences. 2StrucCompare provides ‘difference information’ at distinct macromolecular levels; it calculates and visually displays overall fold differences (an RMSD analysis) but additionally, and at the heart of the package, it can identify differences between conformations at the secondary structure and at the residue level, such as contact differences or side chain conformational differences found between the two chains. By combining tertiary structure alignment with a meta-analysis of per-residue secondary structure assignment and residue structural differences, 2StrucCompare captures both the large differences and subtler differences found between structures. It can therefore enable analyses of the hinging and twisting of protein domains at the macro level, for example, whilst also providing an analysis tool to explore side chain conformational changes that could arise from allosteric interactions or result from an external excitation such as a light-inducing conformational change at the atomic level. This package can therefore provide the visual evidence of the subtle differences between protein structures that could be key to explaining crucial steps involved a proteins’ function.

## MATERIALS AND METHODS

The paired protein chains are aligned by their tertiary structures using TMalign (3) and their sequences aligned by the Needleman-Wunsch global alignment method implemented in the Bio.PDB BioPython library (10,11). Their secondary structures are then determined according to four established methods: DSSP (12,13), STRIDE (14), P-SEA (15) and STICKS (16). As each method employs different strategies for assigning secondary structure elements, small conformational differences between paired residues can result in different secondary structure assignments to these pairs, and the user may choose to utilize each or any of these methods. Should any method fail to produce an output it is disabled as a choice for the user.

2StrucCompare has been implemented as a fully browser-based web application, with no installation or login required. The frontend webserver presented to the user is written in JavaScript, taking advantage of the open source NGL WebGL molecular visualization library (17). The backend of the webserver uses PHP and Python to control file input/output and validation and to perform the initial analyses of the protein structures. The outputs of the four different secondary structure assignment programs, sequence alignments and all structural alignments are calculated on the server and then presented using the aforementioned JavaScript frontend, where comparative analyses of the structures are performed dynamically as the user interacts with the webpage interface. A more extended methodology is described in the Supplementary Data.

## INPUT AND OUTPUT

### Input

The user may upload PDB- or mmCIF-formatted files and/or supply PDB codes for obtaining from the RCSB protein data bank (18) (www.rcsb.org). Each submission or job is assigned a random universally-unique identifier to obfuscate the results URL for security reasons while still allowing the user to bookmark or share the results they obtain if they wish. Using a pull-down menu, the user can pair chains from their chosen proteins and both proteins are visualized using the NGL library (17) to assist this process. The package is also suitable for the analysis of structures obtained from molecular dynamics (MD) trajectories by converting the trajectory to PDB format with each frame’s coordinates contained in individual MODEL records. For such PDB files where more than one model structure is present (such as the MD PDB format and NMR files for example), the user can chose from a pull-down menu the models to compare. In boxes adjacent to their chosen chains and models, the user can also fine-tune their choice by selecting a range of residues for each chain. These ranges would then be those that are compared. It is also feasible to load into the two comparison boxes a common PDB code and then chose to compare one subunit against another (obviously only if the structure has more than one polypeptide chain in the file). Similarly, it is possible to compare one sequence range against another within the same polypeptide chain (especially of interest where a protein may have evolved from gene duplication, for example). Protein structures with no defined resolution, such as NMR structures or models, are assigned a value of 2.8 Å as this has been identified as a reliable average for solved NMR structures (19). With all input data now collected, the chosen pairs of chains are then submitted to the server where the initial analyses take place. Details of these are in the Supplementary Data. User data that are stored include the calculated alignment features and the output data that are sent to the users’ browser for implementation there. Files generated for an analysis run are stored on the server for a maximum of 48 h only.

### Output

The 2StrucCompare output page is comprised of the ‘Results’, ‘Sequence Viewer’ and ‘Structure Viewer’ sections. The webserver performs all the comparative analyses so changes are made in real time in response to user input. User selection of the chain pairs is made in the ‘Results’ section. A table of statistics on the overall three-state secondary structure content for each chain (‘**Summary of Whole Chain**’) gives percentage content of H (Helix), E (Beta Sheet) and O (‘Other’, in the past often referred to as ‘Random Coil’, which is neither random nor coil in nature). A further term, X, showing in the consensus line, is delineating when no secondary structure consensus is found between the chosen methods in their extended secondary structure state format (see below and the Supplementary Data), then such a residue becomes undefined and designated X. An additional term, the calculated RMSD value between the two structures, defines the degree of ‘similarity’ between the two protein chain Cα traces. A further table (‘**Summary of Selection**’) provides the same information for the currently selected residues (as described below) that is dynamic in content, being modified as the user makes alterations to their chosen residues. Residues deemed ‘missing’ in the chain structures in the PDB files are assigned to the ‘Other’ secondary structure percentage calculations by default, but may be removed from the calculations by unchecking the box provided.

The fully defined eight secondary structure features of DSSP (12) data form the initial default display. This includes the residue sequence for both paired chains, the SEQ line (with designated numbering from the structure files, RESNUM above it), the BFACTORS line, the percentile rank of per-residue average B factors dependent on chain resolution (as defined in the Supplementary Data) and the associated secondary structure CONSENSUS line (only DSSP initially). Between the individual chain data are the DIFFERENCES, CA DIST, SC DIST and CONTACTS lines corresponding with the protein residue sequence lines. Briefly these are; DIFFERENCES, residues having different pairwise assigned secondary structures; CA DIST, the per residue Euclidean distances between aligned residues calculated using their Cα atom coordinates; SC DIST, for identical paired residues it is assigned as the maximum interatom distance of like atoms determined following alignment of their main chain atoms; and CONTACTS, a value obtained from calculation of the union of the paired residues contact sets for both protein chains, followed by subtraction of the intersect to get the number of differences between the residues (see the Supplementary Data). When further secondary structure methods are added to the output, their respective secondary structure lines are added and the CONSENSUS lines are updated in real time to reflect these additions. The DIFFERENCES line is also updated (more in the Supplementary Data).

## CASE STUDIES

The unique versatility of this package can be best illustrated using case studies as examples: comparisons between open [5HVX] (20) and closed [6MWA] (21) structures of a voltage-gated sodium channel and a comparative analyses of time-resolved structures of the photoactive yellow protein (22).

### Voltage-gated sodium channel

**Figure 1.**
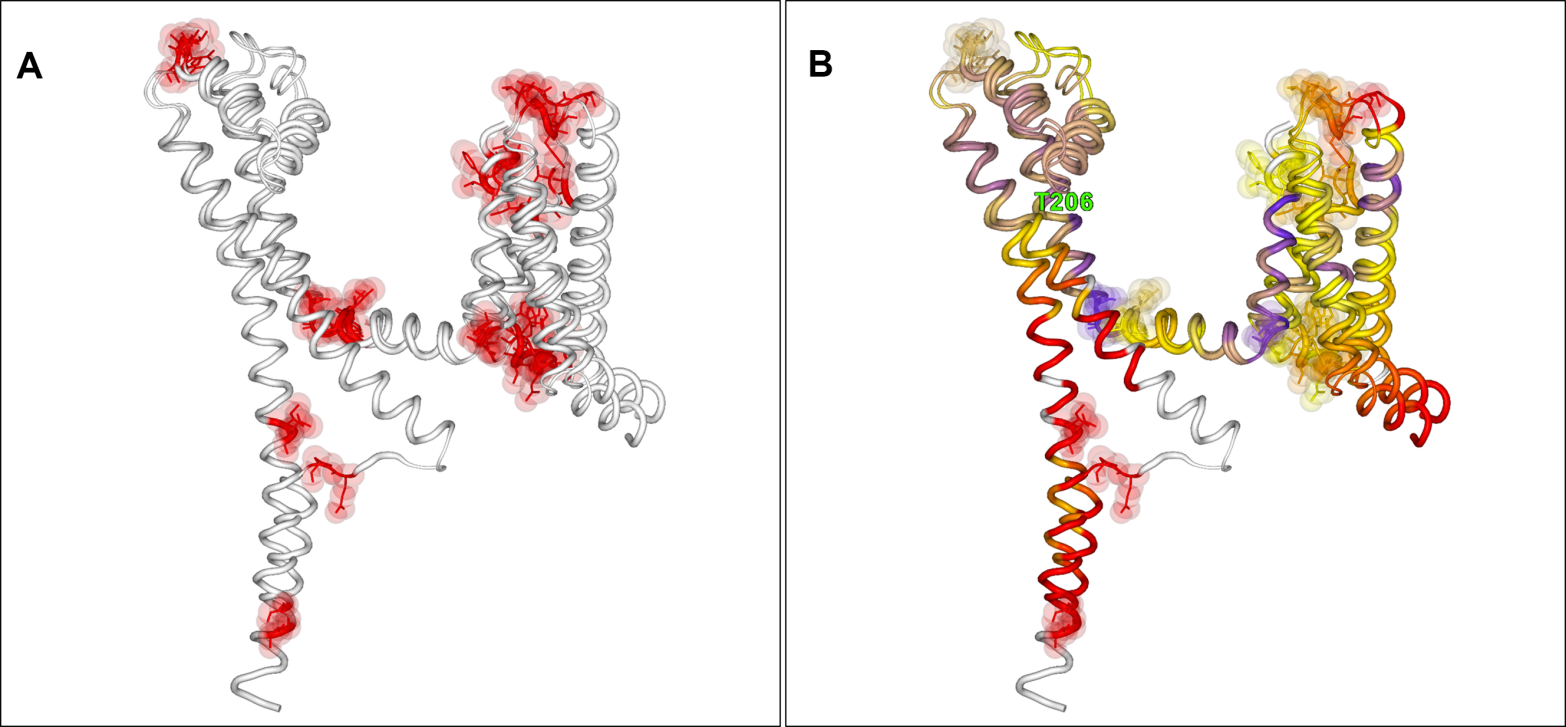
2StrucCompare Structure Viewer screenshot downloads showing the comparisons between open (5HVX) and closed (6MWA) voltage-gated sodium channel structures. (**A**) The secondary structure DIFFERENCES data showing that conformational differences are at the periphery of the structures and at distinct turn regions in the chains. (**B**) The Cα distance output showing the critical hinge at Thre206 where this movement has enabled the structure to be open in the 5HVX structure in comparison to the 6MWA closed conformation.

Figure 1 shows structure comparisons between the NavMs open (20) and NavAb (21) closed voltage-gated sodium channels from *Magnetococcus marinus* and *Arcobacter butzleri*, respectively. Figure 1A shows the differences associated with these structures, where the two chains have differently assigned secondary structures (by DSSP here) mostly at the ends of chains or at discrete bend points. Of note, in Figure 1B the two structures coloured by their Cα paired distance differences show a distinct hinge between the two long helical chains at position Thr206 (numbered according to the 5HVX structure). Here no changes in secondary structure features occur; the change at this point is, unusually, purely a hinge movement away from the centre of the channel pore axis, and it is this that provides the key structural difference between the open and closed states.

### Photoactive yellow protein (PYP)

**Figure 2.**
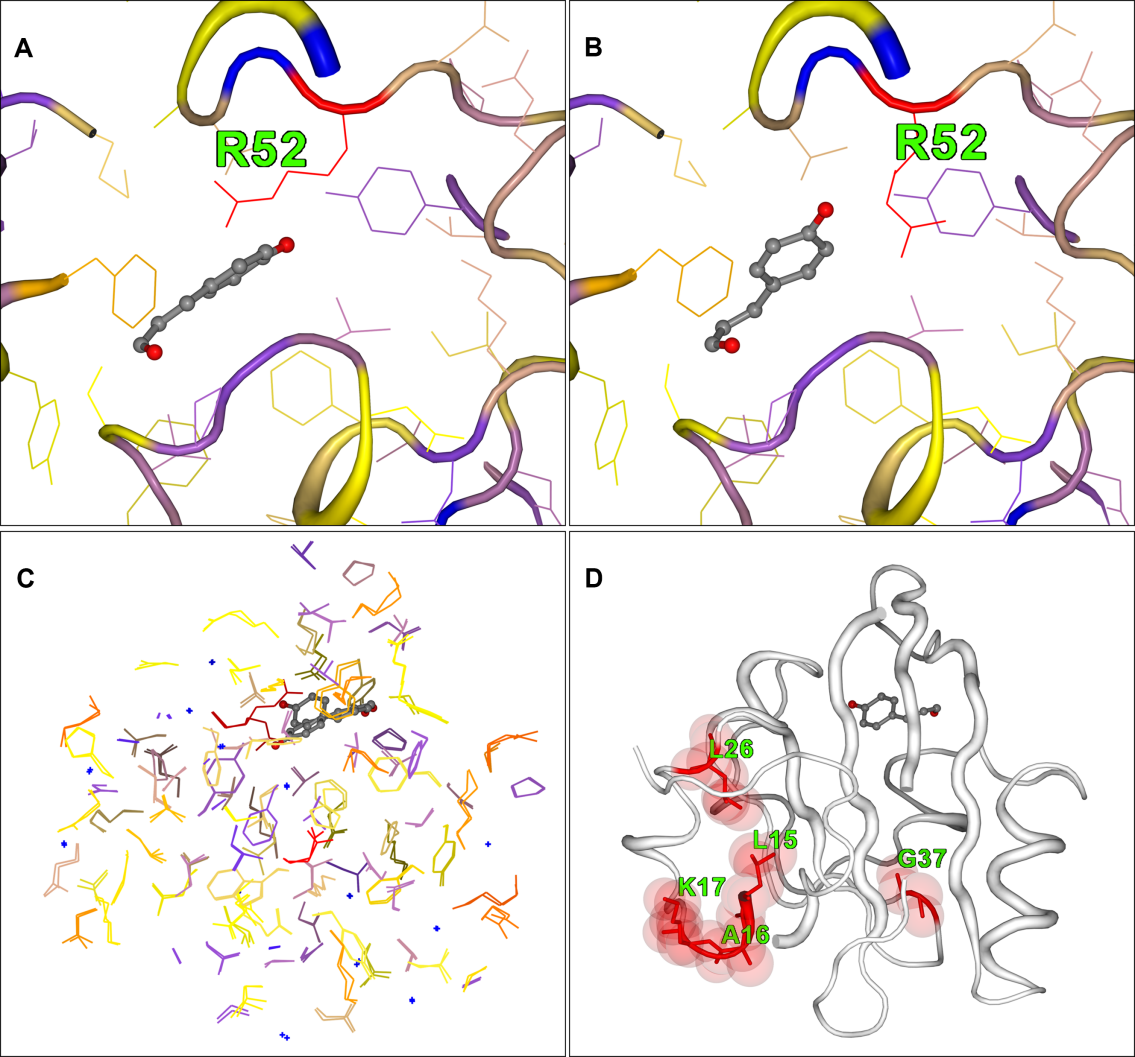
2StrucCompare screenshot downloads for the structure intermediate IL1 (1T1A) from the photoactive yellow protein (PYP) compared with the dark structure (1T18.B). (**A**) The dark conformation of Arg52 adjacent to the chromophore site. (**B**) The IL1 Arg52 conformation where the side chain has been ejected from adjacent to the chromophore. (**C**) A paired view of the side chain only, showing the large numbers of residues that have an increased difference in their positions in the IL1 intermediate from that of their initial dark positions. (**D**) The differences (‘diff’) representation of 2StrucCompare showing the positions of difference between the assigned secondary structures of the dark and IL1 states. This indicates that small conformational changes are already arising in residues near the N-terminal of the IL1 intermediate as a result of the photoexcitation.

The photoactive yellow protein (PYP) from *Halorhodospira halophila* has been studied for many years as a model system for investigating the effects of light-induced excitation of the 4’-hydroxycinnamic acid chromophore that generates conformational changes in the protein structure. A series of five intermediate structural states, labelled IE1, IE2, IL1, IL2 and IL3, were identified using time-resolved Laue crystallographic data collected over a complete photocycle of an E46Q mutant protein by Rajagopal *et al.* (22). Over the entire timescale the protein backbone conformation moves very little; 2StrucCompare calculates a maximum of 0.18Å RMSD between the dark and intermediate conformations during the photocycle. However, side chain movements occur over the whole cycle, propagating initially around the chromophore site. Figure 2 illustrates how 2StrucCompare was utilized to provide information on one particular intermediate state, the IL1 [PDB:1T1A]. In the dark state structure prior to irradiation, the side chain of Arg52 is adjacent to the chromophore (Figure 2A). Subsequently, in the IL1 state the Arg has been substantially moved away from its original position, as indicated by its red colour (Figure 2A and B). In Figure 2C there are a number of side chains that are indicating that they are shifted from their original dark state positions, these being yellow, orange and red in colour. Indeed, it is interesting to note that 12 of the 15 aromatic residues in the structure have different positions associated with them in this intermediate from the dark state. Changes associated with the backbone conformation have propagated towards the N-terminal residues, the extreme of the protein opposing the chromophore position as shown by the differences shown in Figure 2D. The full range of these intermediates in their structure differences for Arg52 relative to the dark state is shown in Supplementary Figure S1.

Of further interest are the data in the Supplementary Figure S2 that show the series of differences in the positions of the side chains that arise between each of the intermediate states. This shows a progressive increasing movement from one state to the next whereby the side chains move farther away from their previous positions in progressing through the intermediate states, then towards the latter intermediate states, near the end of the photocycle, returning to positions more akin to that of their dark state. It is suggestive from the 2StrucCompare appearance that the extra movements in these intermediate positions, particularly when transitioning from the IE2 to the IL1 state, is one way in which the protein is dissipating the extra thermal energy gained from the photoexcitation event.

## DISCUSSION

2StrucCompare provides the user with a ready means to discern the subtle differences between selected pairs of chains from two similar proteins. This can be seen from the macro level of information provided by the overall RMSD difference between the two chains, down to the finer details, where changes identified are at the secondary structure level, where the two chains differ in their assigned structures, and on to the atomic level such as changes in contacts or side chain positions between residues. The information provided through this novel user-friendly package provides a powerful way of gaining a greater insight into just what differences between protein conformations might be key to their functions.

## Supplementary Material

gkz456_Supplemental_Files
