# Supplementary material for: 2StrucCompare: a webserver for visualizing small but noteworthy differences between protein tertiary structures through interrogation of the secondary structure content
Source: Nucleic Acids Res. 2019 May 22;47(W1):W477–81. doi: 10.1093/nar/gkz456 (PMC6602421; doi:10.1093/nar/gkz456)
Supplement: gkz456_Supplemental_Files [file gkz456_supplemental_files.pdf]

2StrucCompare: A webserver for visualizing small but noteworthy differences between protein tertiary structures through interrogation of the secondary structure content

Elliot D. Drew and Robert W. Janes*

School of Biological and Chemical Sciences, Queen Mary University of London, Mile End Road, London, E1 4NS, UK

To whom correspondence should be addressed: r.w.janes@qmul.ac.uk

SUPPLEMENTARY INFORMATION

EXTENDED METHODS

Aligning paired protein chains

For a chosen pair of protein chains, two alignments are made, a global sequence alignment and a structural alignment, the latter being performed using TMalign (1). The sequence alignment performed in 2StrucCompare is by the Needleman-Wunsch global alignment algorithm as implemented in the Bio.PDB BioPython library (2, 3). In order to determine the best approach for sequence alignment, comparisons were made between a global alignment using BLOSUM62 with a gap opening penalty of -11, and a gap extension penalty of -1, to determine the scoring of the alignment, and a simpler approach where the score was calculated using a match = +2, mismatch = -0.5, gap opening penalty = -1, and gap extension penalty = -0.1, where no substitution matrix was used. The BLOSUM62 protocol aligned more distantly related sequences better than the simple method (as expected due to the more nuanced approach when it comes to scoring). However, this posed an issue as it would sometimes lead to this alignment being chosen where visual inspection clearly showed that the TMalign structural alignment was more useful and informative for the comparison. This occurred mainly with sequences which had regions of very low sequence identity but overall a fairly high degree of structural similarity. This issue did not appear to arise with the simpler scoring approach as it was not as efficient on low similarity sequences making it more likely that the structural

alignment would be chosen which generally resulted in a better and more informative per-residue comparison. Since the simpler approach worked as efficiently as required to produce the desired results for this specific use, it was the method chosen over an approach incorporating BLOSUM62.

The best alignment out of the sequence and structural alignments is determined by comparing them through a further gap penalty process. Here, opening a gap results in 1 point and extending a gap results in 0.1 point for either alignment method. The lowest scoring alignment is then used to map secondary structure assignments for display in the Sequence Viewer on the output page, and for the further calculations of C alpha distances, Side chain distances and Contacts differences. The PDB file containing the structural alignment output by TMalign is used for displaying the chains in the Structure Viewer on the output page if this is the best of the two alignments. If, however, it is the sequence alignment that proves the best then a further run of TMalign is undertaken with that sequence alignment used as the input guide to produce the corresponding structure alignment which is then used in the Structure Viewer.

Calculating the Consensus secondary structure

After determining the best alignment, the four secondary structure methods for the paired chains are calculated. The consensus is then calculated for each residue by “majority rule”. If 50% or more of the assigned secondary structure classes are the same from each method that assignment becomes the consensus for that residue. If there is no clear majority, the assignment is considered to be undefined for the consensus sequence, indicated by an "X" in the CONSENSUS line as described in the main text.

Calculation of the differences in paired secondary structure

A difference between paired secondary structure assignments is represented by a value between 0 (full agreement) and 1 (full disagreement) for each residue, indicating the proportion of secondary structure assignment agreement. This value is recalculated dynamically when adding/removing individual methods. We can explain this with the following two examples.

a) b)

chain A	**Seq**	**AGDFE**	chain A	**Seq**	**FDQHT**
	**DSSP**	**HHHHH**		**DSSP**	**TTTTT**
	**STRIDE**	**TTTTT**		**STRIDE**	**HHHHH**
	**Diff**	**00000**		**Diff**	**111½½**
chain B	**Seq**	**AGDFE**	chain B	**Seq**	**FDQHT**
	**DSSP**	**HHHHH**		**DSSP**	**HHHTT**
	**STRIDE**	**TTTTT**		**STRIDE**	**TTTTT**

For a) above there are NO differences to report because while DSSP and STRIDE assign different secondary structure features for the sequence in chain A, those SAME features are assigned to the same residues in chain B; hence no differences in assignments are seen BETWEEN the chains, and DIFF is 0 (full agreement) for each residue. In b) the case is not the same. Now the secondary structures assigned for chain A by DSSP are all T, while in chain B the first three are DIFFERENT from those of chain A, they are H. For STRIDE in chain A they are all H, while for chain B they are all T; they are ALL DIFFERENT in this case. For the first three assignments ALL ARE DIFFERENT between the chains and so the difference assignment for this part of the sequence would be 1 (full disagreement). For the last two residues, only STRIDE shows differences between the assignments, those for DSSP are identical. So for these last two residues a DIFFERENCE value can be calculated as ½, since only 1 (numerator) of the two methods (2 denominator) in use shows differences. This is reflected in the associated colour scale shown for the DIFFERENCE line in the Sequence Viewer and in the Structure Viewer; red being full disagreement, with orange to yellow moving towards greater agreement between paired results with black being 100% agreement. The raw difference values between 0 and 1 are available in the ASCII .csv file of results downloadable from the output page.

Calculation of paired C-alpha distances

The term CA DIST is the per residue Euclidean distance in Ångstroms of aligned residues and is calculated using the C-alpha coordinates of the aligned chains as:

$$d_n = \sqrt{(x_n - x_{n'})^2 + (y_n - y_{n'})^2 + (z_n - z_{n'})^2}$$

where (x_n, y_n, z_n) and ($x_{n'}$, $y_{n'}$, $z_{n'}$) are the orthogonal coordinates for the *nth* paired residues. This is visualised both in the Structure Viewer and in the CA DIST line using a blue-yellow-red colour scheme where blue indicates small deviation and red indicates a deviation >5 Ångstroms. As with the DIFFERENCES line, this can be seen in the Sequence Viewer and in the Structure Viewer when applied to the protein visualisation.

Calculation of the side chain differences distances

Side chain distances are only calculated if the paired residues are the same in both chains. Considering the *nth* residue pair, the method finds the centroids of two groups; the coordinates of the main chain carbon, C alpha and nitrogen of the first chain, and likewise of the second chain. Then a rotation/translation matrix is calculated that would bring the first group into alignment with the second, and said matrix is then applied to all the atoms of the residue in the first chain. Distances are then calculated between equivalent side chain atoms in the residues of the pair. These distances are corrected for when atoms that are equivalent "within" a residue, such as the oxygen's of the carboxyl group of an aspartate for example, might be inverted between the chain pairs, which would otherwise give false distance measurements. Then the calculated maximum inter atom distance is taken to be the side chain deviation distance for that aligned residue pair. In 2StrucCompare, these differences between the side chains are reported on a scale always dependent on the lowest resolution structure of the pair. Reported differences are on a "dynamic" scale therefore, dependent on the structure with the lowest resolution. In order for a difference to be coloured "red" the measured SC DIST has to be equal to or larger than 75% of the lowest resolution. This means that the presence of a low resolution structure in the pairing requires larger differences between their side chain positions before they are coloured "red", by comparison to structures where the chain resolutions are significantly better.

Calculation of the contact differences

In general, using the *nth* residue as an example, the contacts each residue makes within its parent chain are calculated using the NeighbourSearch function in the Bio.PDB Python library (2,3). This function finds all residues within a chosen radius (here 4 Ångstroms) of a given query position. The coordinates of all the atoms of each of the *nth* residues are used as these

“query positions” which generates two contact sets, c_n and $c_{n'}$ respectively. Once the contact sets are calculated, the difference in contacts made (i.e. the non-equivalent contacts in both sets) is determined by counting the number of contacts left after taking the union of c_n and $c_{n'}$ and subtracting the intersect of c_n and $c_{n'}$.

Residue Temperature (B) Factors.

The expected range for temperature (B) factors associated with proteins varies according to the resolution of the structure; the lower the resolution usually the higher the associated B factors for the residues, and the poorer defined regions of structures would also have residues with high B factors. This means that paired residues with higher B factors would need to display larger differences between their side chains for that difference to be significant. For this reason, we chose to put the values for these B factors into a common “percentile” format associated with the resolutions of the structures as follows. We obtained the resolutions and average B factors of every PDB file with resolution range >1.0 Å (as a lower limit) and <=3.5 Å (as the upper limit) and then calculated the average and standard deviation of B factors for all structures across the resolution range in 0.01 Å increments. This gave us information for 99050 structures after culling of structures with no average B factor, and no resolution. We chose a cut off of above 1.0 Å as there were insufficient data points below that to make this region usable. We then produced two polynomial fits for this data, one for average B factor versus resolution and one for standard deviation of B factor versus resolution, in the form:

$y = ax^2 + bx + c$.

In order to generate scaled B factor outputs for pairs of structures we take the resolutions of our inputted chains and calculate what the mean B factor and standard deviation of B factor should be for both of them. (Note that for resolution <1.0 Å the calculation is as if the resolution is 1.0 Å, and if it is >3.5 Å then the calculation is as if it were 3.5 Å). Assuming a normal distribution of B factors around each mean calculated at each resolution, we then take the B factor for each residue to be the average across the residue atoms. We then calculate:

diff = | B factor – mean |

z score = diff / (std dev)

We take this z score and then convert this to a percentile from reference to a standard table (one sided) of z scores. For each residue we essentially have how "far" it is away from the mean of a normal distribution of B factors at the given resolution for the chain. We have therefore produced the percentile rank of the B factors for each residue for its given frequency distribution, such that we can say where it ranks across the range for that structures' resolution. We then colour them according to a rainbow colour scheme where "green" is the average for that resolution, "blue" represents the 0th Percentile of that range, and Red the 100th Percentile of that range. Any residues on the blue colouring side of the scale are better represented in their positions than those on the red side and this should be taken into account when considering differences in side chain positions.

Modifying the Sequence Viewer

3 State Representation

The secondary structures presented in the Sequence Viewer can be modified to a 3-state (Helix, Sheet, Other) representation by checking the associated "**3 state (H,E,O) representation**" box. This alters the appearances in all the secondary structure methods to present their assignments in the three state mode; adding alpha and 3_{10} helices components to be "Helix", strand to be "Sheet" and all remaining components to be summed to become "Other". Changes to the DIFFERENCES and CONSENSUS lines are made in real time, as are any appearance changes in the Structure Viewer.

MiniMap Sequence View

In the default appearance the Sequence Viewer displays ~65 residues in the paired protein chains at any time (dependent on screen size). A scroll bar below the sequence enables the traversal of the entire length of the sequences. To make it easier to see larger amounts of sequence at any one time (albeit with a loss of discernible resolution of the information) the user can check the "MiniMap Sequence View" box. This extends the visible range of the sequence to ~230 residues; however, all the data lines in the Sequence Viewer retain their linked modifications to the Structure Viewer detailed below.

Links between the Sequence Viewer and the Structure Viewer

A powerful feature of 2StrucCompare are the links between the Sequence Viewer and Structure Viewer; modifications made to the data in the former are transferred in real time to the latter enabling a greater ease of interrogation of the structure being viewed.

Select desired residue range: For this section of the Sequence Viewer there are two linked components which in themselves are also linked to the remaining data in the Sequence Viewer and to the Structure Viewer. The first is a two handled bar which can remove residues from the N- and C- termini of the sequences both from view in the Structure Viewer and from any of the calculations. This can be performed by holding down the left mouse button with the cursor over the chosen handle, then moving it towards the bar middle which removes residues from the related chosen sequence termini (left handle – N-terminus, right handle – C-terminus). The second is formed of paired sequence boxes for the two protein chains where values may be input to perform the same truncation procedures. This is an effective way of truncating protein chains, especially of use when potentially considering only information pertinent to a protein domain for example. Both methods of truncation cause the sequence chain information to be "greyed out" for that removed section, designating that region no longer to be in any of the current processes. The sections of the chains that remain showing are termed the "Selection" and the secondary structure data for only these residues are calculated and displayed in the "**Summary of selection**" table. As detailed below, further modifications to the Selection can be made on the sequences and such changes will also grey out the residues. However, the range bar (and the linked paired sequence boxes) overrides any other actions attempted specifically on these truncated termini regions (the greyed out residues). Moving the handles back to their origin positions regains these greyed out residues, making them live once more and adding them back into the calculations.

SEQ: Using the SEQ line on either chain it is possible to remove a range of residues or a specific residue from the Selection. A range may be removed by holding down the left mouse button with the cursor over a chosen residue at the beginning of the range then moving along the line to the end of the chosen range then releasing the mouse button and moving off the line to effect the process. The residues in the range will highlight green during the process and will then grey out and also disappear in the Structure Viewer, additionally being removed from the calculations. Highlighting these residues again will bring them back into the Selection. To remove a single residue, the user may move the cursor to over that residue in the SEQ line and then click the left button. The residue will then grey out and be removed and the process can be reversed in like fashion.

BFACTOR/CONSENSUS/DSSP/STRIDE/PSEA/STICKS: It is possible on the BFACTOR and any of the secondary structure lines associated with each of the methods (DSSP, etc) and also the CONSENSUS lines for each chain, to centre the protein in the Structure Viewer window to a chosen residue. This can be achieved by moving the cursor to over any one of these lines at which point a "tool tip" will indicate which residue it is, and then by clicking the left mouse button the structure will move to centre around that residue in the Structure Viewer.

Inverting the difference selection: The user can invert the current DIFFERENCES selection such that those currently displayed are switched off, and those off are switched on. This may be done by clicking on the **Invert diff. Sel** button.

Toggle all differences OFF/ON: The user may also wish to switch on/off all the differences which is accomplished by clicking on the "**Toggle all diff. OFF/ON**" button. Here either OFF or ON shows dependent on the action the button will perform.

Reset selection: Clicking this button reloads and resets the loaded paired protein chain data; the view orientation of the protein pair in the Structure Viewer is retained and not reset, but the pair of proteins are re-centred and made fully visible.

Download results: Clicking this button downloads a .csv file containing all the available calculated data for the two currently chosen protein chains.

The Structure Viewer

The default display for the Structure Viewer is of both the first two chosen overlaid protein chains in the "diff" colouring scheme with the DIFFERENCE residues of DSSP default showing in red with a semi-transparent surface surround. Various check boxes are present adjacent to the Structure Viewer window. The difference side chains can be switched off by unchecking the "**Difference Sidechains?**" box. The backbone view(s) can be removed by unchecking the "**Backbone Visible?**" box, and any ligands in the structure(s) can be removed from view by unchecking the "**Ligands?**" box. The protein chains can be viewed in different colour schemes by choosing from a pull down menu. These are: **diff**, **ca_distance**, **sc_distance**, **contacts, bfactors**, **dssp**, **stride**, **psea**, **sticks**, **chain** or **cpk**. A choice of chains to display can be made from another pull down box menu: **Both**, "**chain 1**", "**chain 2**" where these latter two indicate a shortened named identifier for the two chosen proteins.

The displayed protein chain(s) in the Structure Viewer can be moved using various mouse features (illustrated in this instance by a two button, central wheel mouse as an example). Holding down the left button enables rotation, whilst holding down the right enables translation. Rotating the central wheel with cursor within the Structure Viewer provides a zoom in/out to the display. Hovering the cursor over any atom brings up its sequence information, and holding down the Ctrl key then clicking the left mouse button will label that residue at that atom position with its one letter code and sequence position for the chosen chain. All labels are chain-specific and are retained for that chain only. For clarity in the Structure Viewer, labels may be increased in size from the default to "**x2**", "**x4**" and "**x6**" by subsequent left clicking the "**Label x 2**" button, (where the next action of that button replaces that which has been undertaken by the click); a further click reduces the size back to the default. Labels will also increase in size with the zoom function. Additionally, all labels whether in view or not, may be switched off from the Structure Viewer by left clicking in the "**Remove Labels**" button. Clicking the central button (illustrated again by the central wheel in this instance) whilst hovering the cursor over any atom will centre the view on that atom in the Structure Viewer. The user may centre the entire chain (regardless of whether it is in view at the time) by left clicking the **center** box. Any graphical view of interest can be produced as a downloaded picture by left clicking the "**screenshot**" box. This produces a .png file with transparent background so the user may then choose their background for subsequent presentation.

2Struc

In addition to the 2StrucCompare package there is an updated version of 2Struc: the secondary structure server (7). All of the features presented as actions on a single protein chain in 2StrucCompare are available in this improved version of 2Struc with the exceptions that no side chains can be visualised and the backbone cannot be removed from the viewer image.

Supplementary References

1. Zhang,Y. and Skolnick,J. (2005) TM-align: A protein structure alignment algorithm based on TM-score. *Nucleic Acids Res.*, **33**, 2302-2309. PMCID: PMC1084323 DOI: 10.1093/nar/gki524

2. Hamelryck,T. and Manderick,B. (2003) PDB parser and structure class implemented in Python. *Bioinformatics,* **19**: 2308–2310. PMID: 14630660 DOI:10.1093/bioinformatics/btg299

3. Cock.P.A., Antao.T., Chang,J.T., Chapman,B.A., Cox,C.J., Dalke,A., Friedberg,I., Hamelryck,T., Kauff,F., Wilczynski,B. and de Hoon,M.J.L. (2009) Biopython: freely available Python tools for computational molecular biology and bioinformatics. *Bioinformatics*, **25**, 1422-1423 PMCID: PMC2682512 DOI: 10.1093/bioinformatics/btp163

4. Kabsch,W. and Sander,C. (1983) Dictionary of protein secondary structure: pattern recognition of hydrogen-bonded and geometrical features. *Biopolymers*, **22**, 2577-2637. PMID: 6667333 DOI: 10.1002/bip.360221211

5. Touw,W.G. Baakman,C., Black,J., te Beek,T.A.H., Krieger,E., Joosten,R.P. and Vriend,G. (2015) A series of PDB-related databanks for everyday needs. *Nucleic Acids Res.*, **43**, D364-D368. PMCID: PMC4383885 DOI:10.1093/nar/gku1028

6. Frishman,D. and Argos,P. (1995) Knowledge-based protein secondary structure assignment. *Proteins*, **23**, 566-79. PMID: 8749853 DOI: 10.1002/prot.340230412

7. Klose,D.P., Wallace,B.A. and Janes,R.W. (2010) 2Struc: the secondary structure server. *Bioinformatics*, **26**, 2624-2625. DOI: 10.1093/bioinformatics/btq480

Supplementary Case Study Figures

Figure Legends

Figure S1. The dark state (pdb code: 1T18 second model) and the intermediate states named IE1, IE2, IL1, IL2 and IL3 (pdb codes: 1T18 first model, 1T19, 1T1A, 1T1B, 1T1C) found in time-resolved Laue crystallographic studies on photoactive yellow protein (PYP); side chain positions (coloured by SC DIST values) adjacent to the chromophore site in the protein. a) the dark state of the protein (all plates are in relation to the side chains in a); b) IE1 state relative to that dark state. The Arg52 side chain is orange coloured indicating a notable movement away from its original dark position. c) IE2 relative to the dark state. This state appears "closer" to the original dark position (being more yellow than orange) but this is because the side chain is beginning to twist round which means its distance measurement comes out slightly closer to that of the dark state. d) IL1 relative to the dark state. In this state the Arg52 side chain has been ejected from the chromophore neighbourhood as indicated by the red colour. e) IL2 relative to the dark state. Again the Arg52 is coloured red indicating a significant movement from its original position. f) IL3 relative to the dark state. The photocycle has completed by this time around the chromophore site and the Arg52 side chain is returning to the original dark position as indicated by the more purple colour.

Figure S2. The differences found between the structures as PYP changes from one intermediate structure to the next, coloured as side chain differences in positions on the main chain structure. Colouring of red indicates substantial relative differences in side chain positions from the previous state. a) The dark state to IE1 change. Most side chain differences are in the yellow range of movement from the previous state. Some have moved away further (into the orange) and a few have made sufficient movement to be coloured red. b) The IE1 to IE2 change. This change initiates a more substantial degree of difference from the previous state where more residues have now coloured to orange and to red. c) The IE2 to IL1 change. This transition is the one where the largest number of residues have made substantial movements away from their positions in the previous state. It is almost suggestive of the increased energy imparted to the system by the photoexcitation being dissipated away by increased movements being imparted to many of the residues around the protein. It is almost as if the protein is using the motions of the side chains to "shake off" the excess energy gained from the excitation; a kind of "quaking event". d) The IL1 to IL2 change. Whilst there is still a significant amount of difference in the positions of the side chains relative to their last stage positions, there is clearly less of an extent in this time frame as compared with the last change. e) the IL2 to IL3 change. The numbers of side chains

coloured red here is noticeably fewer indicative that the side chains have now dissipated their possible increased energy into the surrounding medium. f) the IL3 relative to the dark state (as the photocycle is theoretically complete at this point). This is more of a “comparison” than a “transition” to the dark state as the photocycle is considered as over at this point in the time course of the experiment, yet the protein is still in an altered state relative to the dark state. However, it is quite apparent from the lack of any residues in the red state that the IL3 intermediate has very comparable characteristics to that of the dark state although there has been a transfer of overall, subtle structural changes towards the N-terminal of the protein (as indicated in the main text and Fig, 2d).

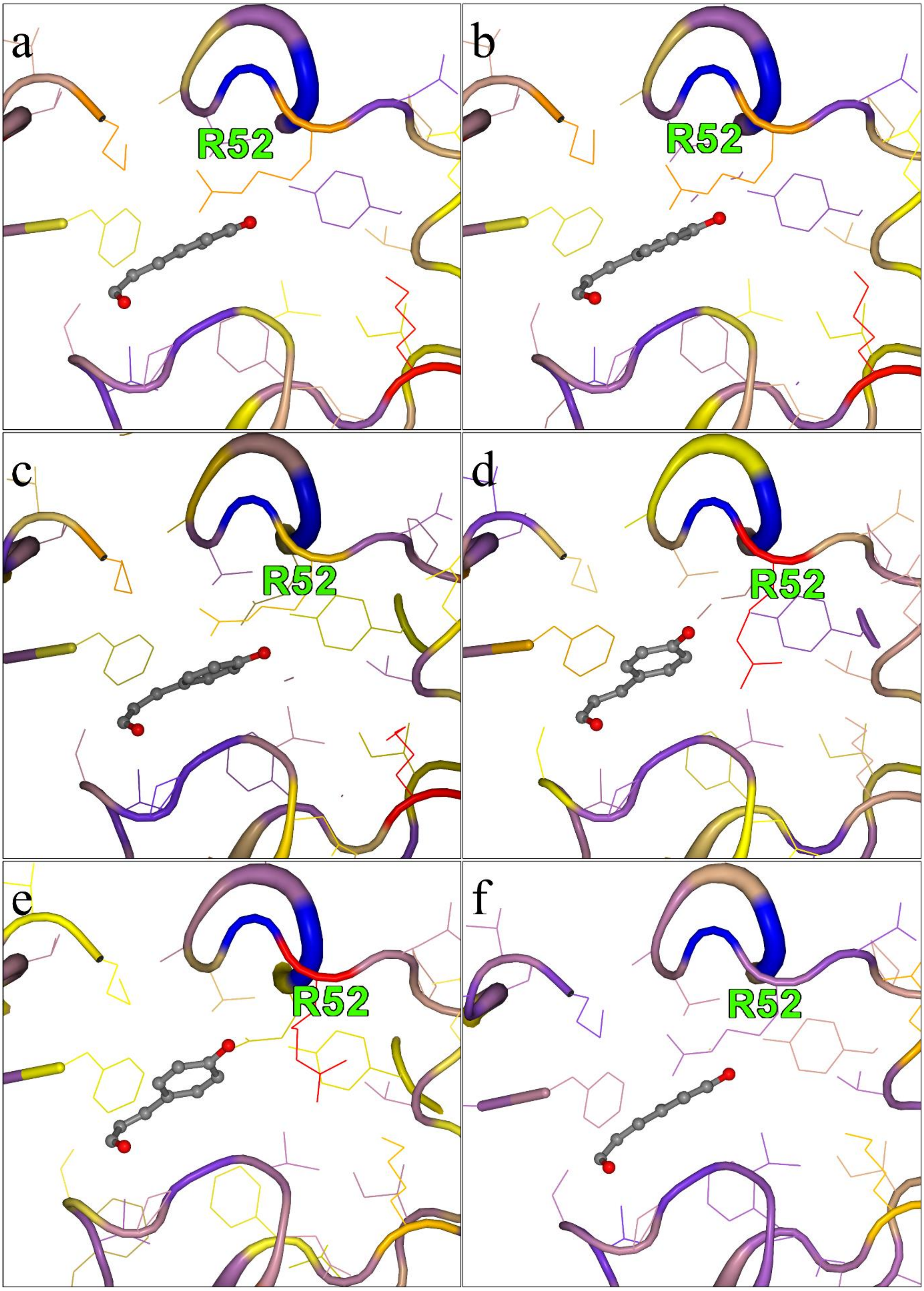
a
R52
b
R52
c
R52
d
R52
e
R52
f
R52

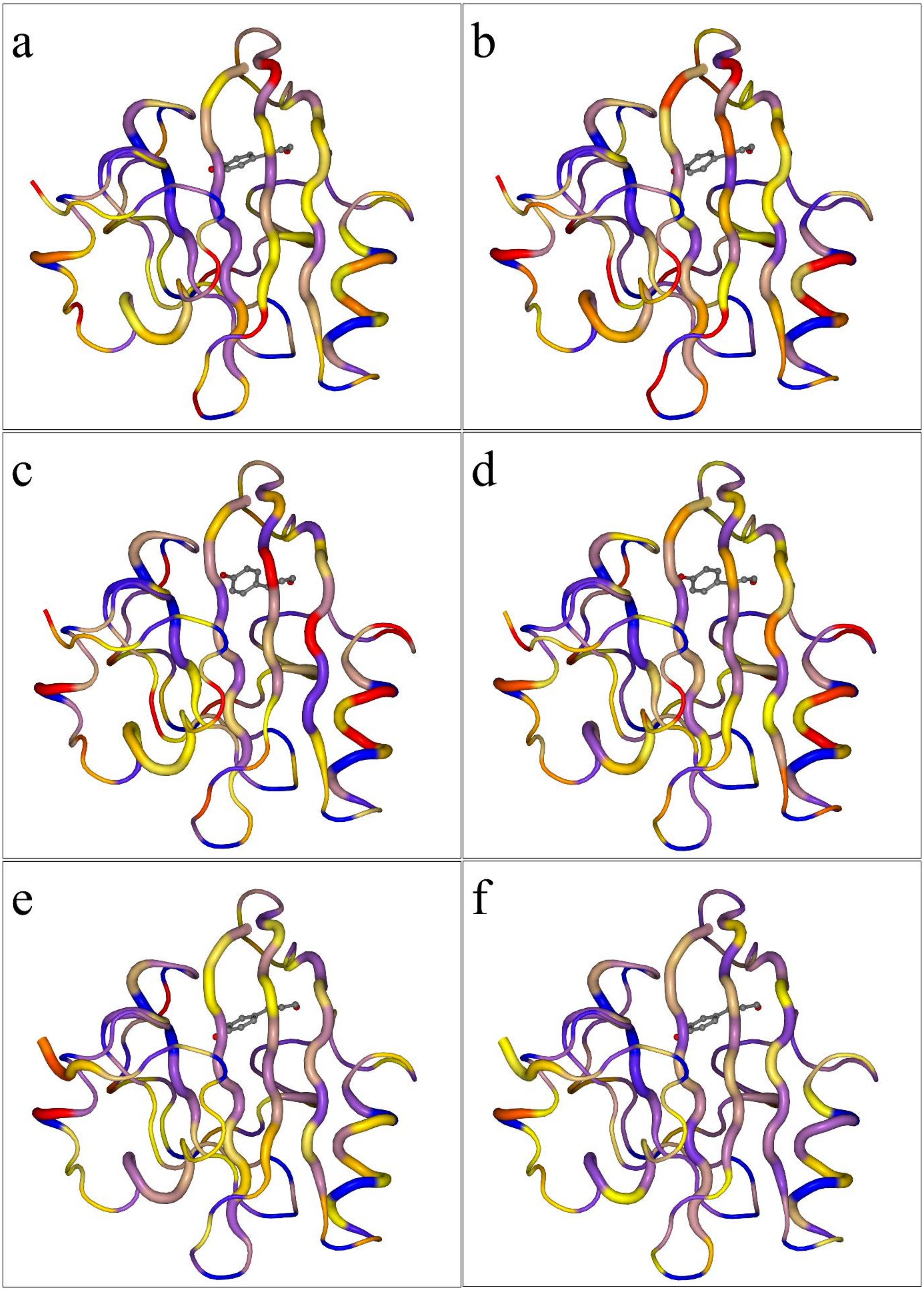
a
b
c
d
e
f
